# Cardiac Magnetic Resonance in HCM Phenocopies: From Diagnosis to Risk Stratification and Therapeutic Management

**DOI:** 10.3390/jcm12103481

**Published:** 2023-05-16

**Authors:** Roberto Licordari, Giancarlo Trimarchi, Lucio Teresi, Davide Restelli, Francesca Lofrumento, Alessia Perna, Mariapaola Campisi, Cesare de Gregorio, Patrizia Grimaldi, Danila Calabrò, Francesco Costa, Antonio Giovanni Versace, Antonio Micari, Giovanni Donato Aquaro, Gianluca Di Bella

**Affiliations:** 1 Department of Biomedical and Dental Sciences and Morphological and Functional Imaging, University of Messina, 98100 Messina, Italy; robertolicordari@gmail.com (R.L.);; 2 Department of Clinical and Experimental Medicine, University of Messina, 98100 Messina, Italy; 3 Academic Radiology Unit, Department of Surgical Medical and Molecular Pathology and Critical Area, University of Pisa, 56126 Pisa, Italy

**Keywords:** hypertrophic cardiomyopathy, cardiac magnetic resonance, phenocopies

## Abstract

Hypertrophic cardiomyopathy (HCM) is a genetic heart disease characterized by the thickening of the heart muscle, which can lead to symptoms such as chest pain, shortness of breath, and an increased risk of sudden cardiac death. However, not all patients with HCM have the same underlying genetic mutations, and some have conditions that resemble HCM but have different genetic or pathophysiological mechanisms, referred to as phenocopies. Cardiac magnetic resonance (CMR) imaging has emerged as a powerful tool for the non-invasive assessment of HCM and its phenocopies. CMR can accurately quantify the extent and distribution of hypertrophy, assess the presence and severity of myocardial fibrosis, and detect associated abnormalities. In the context of phenocopies, CMR can aid in the differentiation between HCM and other diseases that present with HCM-like features, such as cardiac amyloidosis (CA), Anderson–Fabry disease (AFD), and mitochondrial cardiomyopathies. CMR can provide important diagnostic and prognostic information that can guide clinical decision-making and management strategies. This review aims to describe the available evidence of the role of CMR in the assessment of hypertrophic phenotype and its diagnostic and prognostic implications.

## 1. Introduction

The hypertrophic phenotype is a commonly observed finding that can result from various etiologies [1]. Accurate differential diagnosis of cardiomyopathy phenocopies is crucial for appropriate disease management, as highlighted by both the European Society of Cardiology and MOGE classifications [1,2].

While ECG provides only some features of hypertrophic phenotype [3], echocardiography is, currently, the first-line diagnostic tool for identifying myocardial hypertrophy and excluding many loading conditions and/or valve dysfunctions (such as aortic stenosis) [4]. However, cardiac magnetic resonance (CMR) imaging has emerged as the preferred imaging modality for making a definitive differential diagnosis of hypertrophic phenocopies. This is due to its ability to provide tissue characterization through an integrated evaluation of different CMR sequences. CMR offers a higher degree of accuracy and reproducibility for confirming morphological characteristics than echocardiography. In particular, the presence, localization, and extent of late gadolinium enhancement (LGE) on CMR can provide better definition of the etiology as well as prognostic stratification in many hypertrophic phenocopies.

Moreover, CMR is an ideal tool for differential diagnosis of hypertrophic phenocopies due to its multiparametric tissue characterization. In the last 10 years, T1 and T2 mapping have enabled quantitative and detailed analysis of myocardial tissue characterization. Mapping increases not only the diagnostic accuracy, but also provides a quantitative tool for therapeutic management of drugs that modify diseases, particularly in infiltrative cardiomyopathies.

The aim of this overview is to underscore the role of CMR in the diagnosis, prognosis, and treatment of patients with definite and/or suspected hypertrophic phenotype.

## 2. HCM Phenocopies (Similarities and Differences)

The definition of left ventricular hypertrophy (LVH) is not as straightforward as one might assume. LVH can be defined based on the assessment of cardiac mass or based on the measurement of wall thickness. However, as detailed in Section 3.1, each method has its limitations. Given the several possible causes of LVH, cardiomyopathy (HCM or phenocopy) should be suspected in the face of a precise hypertrophy phenotype.

The hypertrophic phenotype refers to the thickening of the left ventricular wall to 15 mm or more in at least one myocardial segment, measured by an imaging technique, and not fully explained by loading conditions (such as hypertension), myocardial ischemia, or valve dysfunction [5]. This phenotype can be caused by sarcomeric and non-sarcomeric cardiomyopathies. Non-sarcomeric cardiomyopathies can be divided into infiltrative diseases, such as CA, and storage diseases, such as AFD, glycogen storage diseases, and mitochondrial diseases [1]. Hypertensive heart disease (HHD) should be considered as another possible HCM-mimicking disease; although it is not strictly a genetic cardiomyopathy, it is common in the general population and it may present some features. Hence, a differential diagnosis should be always kept in mind.

Hypertrophic phenocopies have a prevalence ranging from 1:500 (0.2%) to 1:200 (0.5%) in general population, and sarcomeric HCM represents up to 60% of cases, while CA is the second-most common etiology, followed by AFD [3,4,5,6].

HCM is a global disease and sarcomeric HCM is a leading cause of genetic and heritable heart disease, resulting in a high risk of sudden cardiac death, especially in young people [7,8,9,10]. It is caused by mutations in genes which encode sarcomeric proteins. Generally, patients with a sarcomere protein mutation tend to have more severe hypertrophy, microvascular dysfunction, and myocardial fibrosis [9].

Diagnosis of HCM should be guided by familial history and clinical red flags, which vary for each hypertrophic phenocopy. However, the absence of signs and symptoms does not exclude diagnosis, and, unfortunately, sudden cardiac death can be the first manifestation of HCM. Electrocardiograms (ECG) can support HCM suspicion, and rhythm alterations are frequent, especially atrial fibrillation (AF). Echocardiography shows LVH and diastolic dysfunction as main features. Other typical findings are left atrium (LA) enlargement, systolic dysfunction evaluated by global longitudinal strain (GLS), and left ventricle outflow tract (LVOT) obstruction due to systolic anterior motion (SAM) of the mitral valve [1,5].

Hypertrophic phenocopies cannot be reliably differentiated based on imaging alone. However, cardiac imaging can provide a definite diagnosis in many hypertrophic cardiomyopathies when integrated with an accurate clinical evaluation. As phenocopies are relatively rare, it is crucial to diagnose these conditions at an early stage, as their natural history, management, and prognosis differ significantly from that of HCM.

The main phenocopies could be classified according to etiology as follows [9,11,12,13,14]:

- Glycogen Storage Disorders (GSD): This group includes Danon disease, Pompe disease (GSD type 2), Forbes/Cori disease (GSD type 3), and PRKAG2 cardiomyopathy;
- Anderson–Fabry Disease: This is a lysosomal storage disorder, due to a mutation in the α-galactosidase A gene, and it is an X-linked recessive disease;
- Cardiac amyloidosis: This is due to extracellular deposition of amyloid material, and it can cause infiltrative or restrictive cardiomyopathy. Concentric LVH is observed;
- Mitochondrial cytopathies: This heterogeneous group is caused by mutations of the maternally inherited mitochondrial genome and leads to dysfunctional energy production and multisystemic involvement, especially involving central nervous system, heart, and skeletal system;
- Hypertensive heart disease;
- Athlete’s heart.

## 3. Cardiovascular Magnetic Resonance

Cardiovascular magnetic resonance imaging (CMR) is the reference standard for the reliable and reproducible evaluation of cardiac mass, volume, and function. CMR offers several advantages, such as high spatial resolution, unlimited field of view, and excellent tissue characterization capabilities, which enable the assessment of cardiac anatomy and function, as well as the identification and distribution of hypertrophy and myocardial tissue abnormalities. A comprehensive CMR evaluation of morphological features, LGE, myocardial T1 mapping, and extracellular volume (ECV) allows for both a definite diagnosis and a prognostic stratification. Table 1 outlines differences between CMR findings in HCM and other phenocopies.

### 3.1. Morpho-Functional Features in Hypertrophic Phenotypes

LVH is an independent predictor of future cardiovascular events [15]. The most common method for defining and quantifying LVH is assessing LV wall thickness from an end-diastolic cine picture in the short-axis plane. The normal thickness of the LV myocardium is less than 11 mm [16]. LVH is categorized as mild (11–13 mm), moderate (14–15 mm), or severe (>15 mm). Moreover, increased LV mass is required for a more accurate diagnosis of LVH. Using end diastolic images, this parameter is calculated as the difference between the total epicardial volume and the total endocardial volume, multiplied by the specific density of myocardium (1.05 g/mL). Estimates of LV mass (LVM) are often indexed to body size, providing an LVM index (LVMi) value in g/m^2^ if adjusted for body surface area (BSA) or g/m if corrected for height. Many factors (e.g., age, race, physical activity, hypertension, diabetes, and smoking history) could impact LVM, and various threshold values may be appropriate for different groups [17]. It is important to underline that, despite increasing myocardial wall thickness, the LV mass may remain within normal range, especially when the thickening is focused.

The relative wall thickness (RWT), a ratio generated from LV wall thickness and LV chamber diameter, is commonly used to classify LVH into two patterns. LVH is defined as concentric when the RWT is raised, and as eccentric when the RWT is not increased. This categorization approach has several shortcomings, including its reliance on a ratio of linear dimensions and its failure to account for LV dilation in isolation, a crucial feature of geometric remodeling. Moreover, LVH can be classified in symmetric and asymmetric hypertrophy. The former term should be adopted when a homogenous thickening of several LV segments is seen, while the latter indicates a disproportionated thickening of some LV segments in particular. In the context of an asymmetric LVH, CMR is superior to echocardiography in the identification of morphological alterations in several areas, such as in LV apex, basal anterior wall, and anterolateral wall.

**Figure 1:**
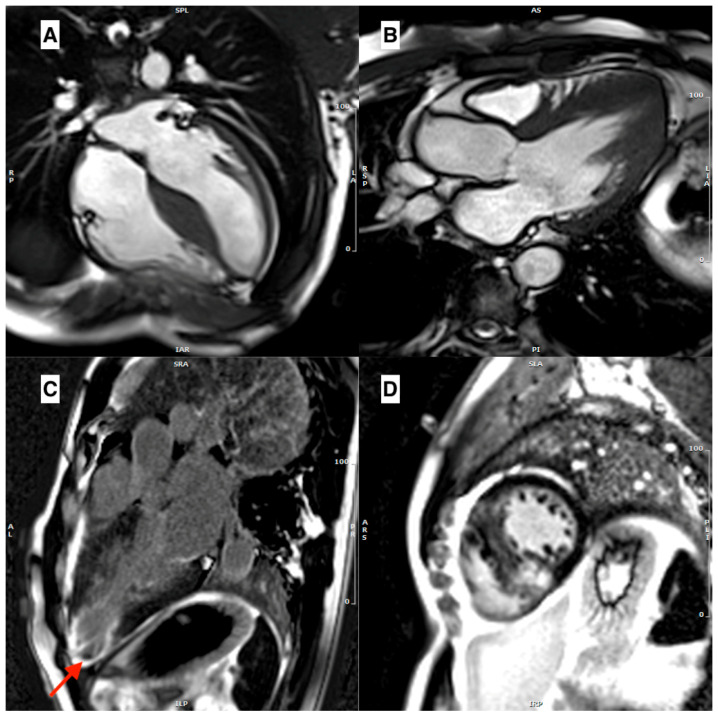
(**A**) A cine-bSSFP 4-chamber image in a telediastole, showing a case of asymmetrical HCM with a thickened interventricular septum. (**B**) A cine-bSSFP 3-chamber image in a telediastole, showing an apical form of HCM. (**C**) An LGE 3-chamber image showing transmural LGE in left ventricular apex with aneurysmal dilatation, with a thrombus inside (red arrow). (**D**) A mid-ventricular short axis LGE image showing mid-wall LGE in the interventricular septum, in particular in the RV/LV insertion points.

***Sarcomeric HCM*** is the prototype of several phenotypical manifestations of hypertrophy with multiple patterns, from the asymmetric septal pattern (the most common, Figure 1A) to other variants such as apical, mid-ventricular (with and without apical aneurysm), and diffuse. The most frequent sites of increased LV wall thickening in HCM are the confluence of the basal septum with the anterior and inferior right ventricle free walls [18]. A considerable percentage of HCM patients have just one or two LV segments with focal regions of increased wall thickness. In apical HCM, the LV cavity is obliterated at the apex, giving the cavity a distinctive spade-like form in long-axis images (Figure 1B). Typically, the apical wall thickness is >15 mm or the ratio of apical-to-basal LV wall thickness is >1.3–1.5 [19]. Moreover, LV apex could be characterized by apical aneurysms (thin-walled, dyskinetic segment of the most distal portion of the LV with a relatively wide communication to the main cavity in diastole). They occur in 2% of patients with HCM and 13% to 15% with apical phenotype [20]. Although the exact cause of apical aneurysm remains unknown, some theories suggest that apical aneurysm could be a result of increased afterload and high apical pressure due to significant pressure gradient from mid-ventricular obstruction, because coexistence of mid-ventricular obstruction and apical aneurysm are reported in many patients [21]. Other possible explanations include small-vessel disease that reduces coronary flow reserve, compression of the coronary artery by increased systolic myocardial wall stress in the hypertrophic segment, decreased coronary perfusion pressure caused by mid-ventricular obstruction, and coronary artery spasm. This reduced coronary perfusion has been reported in stress-perfusion CMR exams, leading to scar tissue formation [22]. Once apical scar is formed, it is more susceptible than normal myocardium to aneurysmal dilatation, due to high intra-ventricular pressure gradients.

The dyskinetic/akinetic aneurysm increases the risk of apical thrombus formation and thromboembolic stroke (Figure 1C) [20]. LVH severity, SCD, monomorphic VT, LV systolic dysfunction, and heart failure have all been linked to apical aneurysms [23].

In individuals with HCM, the increase in myocardial mass could not be restricted to just the left ventricle. A considerable number of individuals with HCM have morphological RV abnormalities, including increased RV wall thickness (>8 mm) in almost a third and an increase in RV wall mass [24]. CMR can easily detect RV muscle structures that can mimic hypertrophy, such as a prominent moderator band.

In addition, recent reports suggest a higher prevalence of crypts (clefts in compacted myocardium that penetrate more than 50% of wall at end-diastole and collapse at end-systole) localized more commonly in the basal inferior septum and inferior LV free wall in patients with phenotype positive HCM, as well as within samples of genotype-positive phenotype-negative HCM [25]. The presence of crypts (especially numerous), anterior mitral valve leaflet elongation, and aberrant trabeculae suggest the existence of sarcomere gene mutations in genotype-positive, phenotype-negative HCM patients [26]. These additional morphological anomalies might aid the diagnostic process, especially when LVH is moderate (wall thickness between 13 and 15 mm).

Approximately one-third of patients with HCM have resting SAM of the mitral valve leaflets that obstructs the LV outflow tract, while another third has latent obstruction only during exercises that alter loading conditions and LV contractility. SAM of the mitral valve usually leads to lack of normal leaflet coaptation and inferolaterally oriented mitral regurgitation during mid-to-late systole (Supplemental Video). In patients with LVOT obstruction, velocity-encoding flow mapping sequences can be used to determine the peak velocity of blood flow through the LV outflow tract; however, this approach is time-consuming and error-prone. Intravoxel dephasing and signal loss due to phase offset errors further complicate the precise characterization of turbulent flow. Finally, gradients and velocities can only be assessed during rest. For these reasons, Doppler echocardiography is the preferred method for quantifying LVOT obstruction [9].

Furthermore, abnormalities of the mitral valve and papillary muscles may contribute to dynamic LVOT obstruction and have important implications for preoperative surgical myectomy planning in selected candidates with LVOT obstruction and symptoms which are refractory to medical management. Mitral valve abnormalities in HCM usually involve leaflets, which are longer than in normal subjects (anterior mitral leaflet > 30 mm, posterior mitral leaflet > 17 mm). Elongated mitral valve leaflets also contribute substantially to the mechanisms responsible for sub-aortic gradients [27]. Morphological alterations of the papillary muscles can be found as well. These abnormalities include an increased number of papillary muscles, hypertrophy, apical displacement of the papillary muscles, and anomalous insertion directly into the anterior mitral valve leaflet with short or absent chordae tendineae [28].

One of the early signs of HCM is diastolic dysfunction, which is associated with myocardial disarray and fibrosis, even in the absence of LVH. Doppler parameters are effective in assessing diastolic function in HCM using echocardiography. However, the measurements of trans-mitral and transpulmonary vein flow parameters using phase-contrast cardiac MRIs are typically lower than those obtained with echocardiography. This difference may be due to the breath-hold phase-contrast sequence having a lower temporal resolution than echocardiography [29]. Other approaches to evaluate diastolic function using CMR were explored, but they are time-consuming and their use in HCM or in other phenocopies was not reported [30]. LA is often enlarged in HCM patients. Volumetric MR quantification is the best approach to assess the LA size, but it may be too time-consuming. Currently, LA quantification is carried out daily by evaluating LA area in cine-bSSFP images (horizontal and vertical long axis) and then indexing it to body surface area (BSA). An area ≧15 cm^2^/m^2^ is used to identify LA enlargement [31].

Concentric LVH can result from systemic hypertension, and it can be challenging to distinguish between HCM and hypertensive heart disease (HHD) in clinical practice. In contrast to HCM, where the left ventricular wall thickness can exceed 16 mm, the wall thickness in hypertensive heart disease is typically less than or equal to 16 mm [32]. Some features explained above, as usually reported in HCM patients or in genotype-positive/phenotype-negative patients, such as myocardial crypts or LVOT obstruction, are occasionally described in HHD [33].

**Figure 2:**
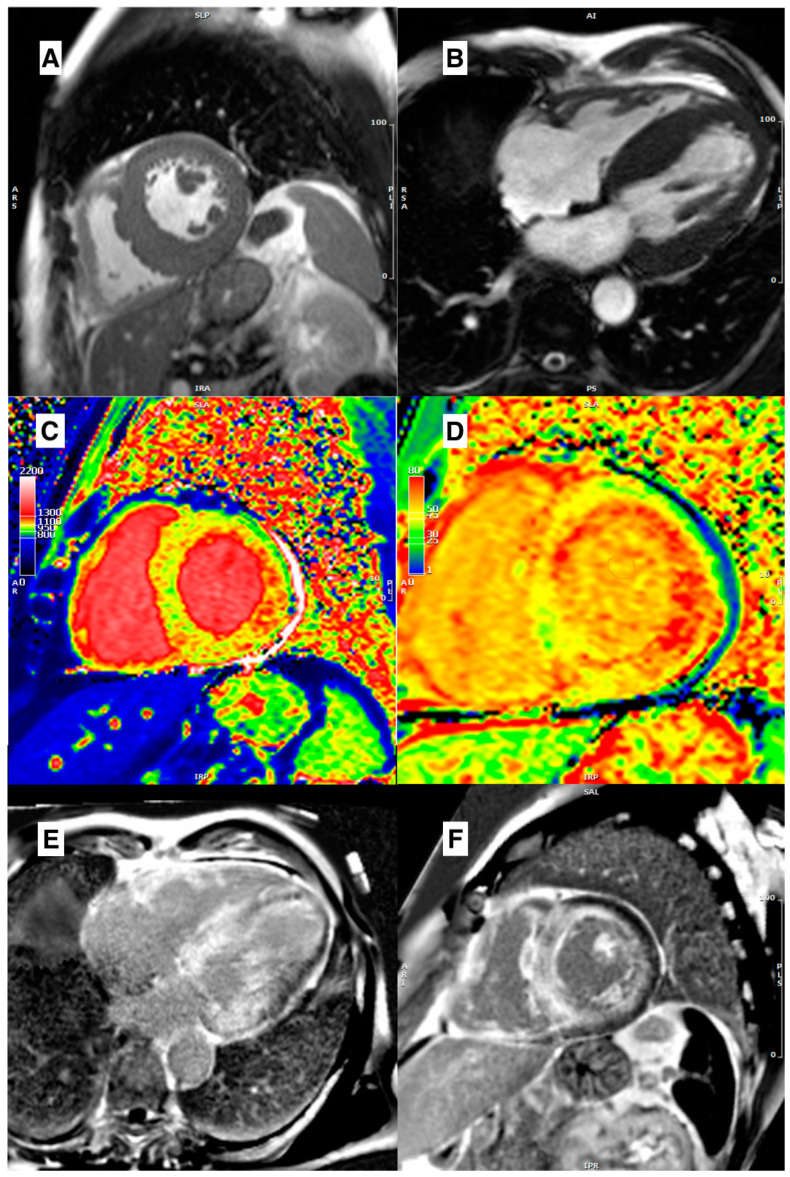
(**A**) Mid-ventricular short axis cine-bSSFP image in a telediastole, and (**B**) cine-bSSFP 4-chamber image in a telediastole, both showing a case of cardiac amyloidosis with asymmetric septal LV hypertrophy. (**C**) Mid-ventricular short axis T1 map showing a diffuse intramyocardial T1 values increase. (**D**) Mid-ventricular short axis ECV map showing a diffuse increase of extracellular volume. (**E**) 4-chamber LGE image and (**F**) mid-ventricular short axis LGE image, both showing diffuse LV and RV LGE with inappropriate myocardial nulling.

Typical morphological features of ***CA*** include LV wall thickening, RV wall thickening, thickening of the interatrial septum and valvular leaflets, and pericardial and pleural effusion [34,35]. LVH is concentric in the majority of patients with CA; however, asymmetric LVH affecting the interventricular septum may also occur (Figure 2A,B) [36]. The LV EDVs are slightly reduced and LV EF is near normal, though it may be decreased, in end-stage disease. In addition to these typical morphological signs, patients with CA can show an increased thickening of “neglected” structures, including crista terminalis and Eustachian valve in the right atrium, or mitroaortic lamina and coumadin ridge in the left one [37]. LA, in CA, is usually enlarged, predisposing patients to a higher incidence of AF. CMR could be helpful in identifying LA enlargement, thickness of the atrial wall and the interatrial septum, and early loss of atrial contraction, despite P wave still evident at ECG.

Occasionally, however, patients with CA demonstrate an atypical pattern of infiltration with asymmetric septal thickening and an LVOT gradient with SAM of the mitral valve that resembles obstructive HCM [38,39,40]. In the same way, myocardial crypts are occasionally described in patients with CA [41].

**Figure 3:**
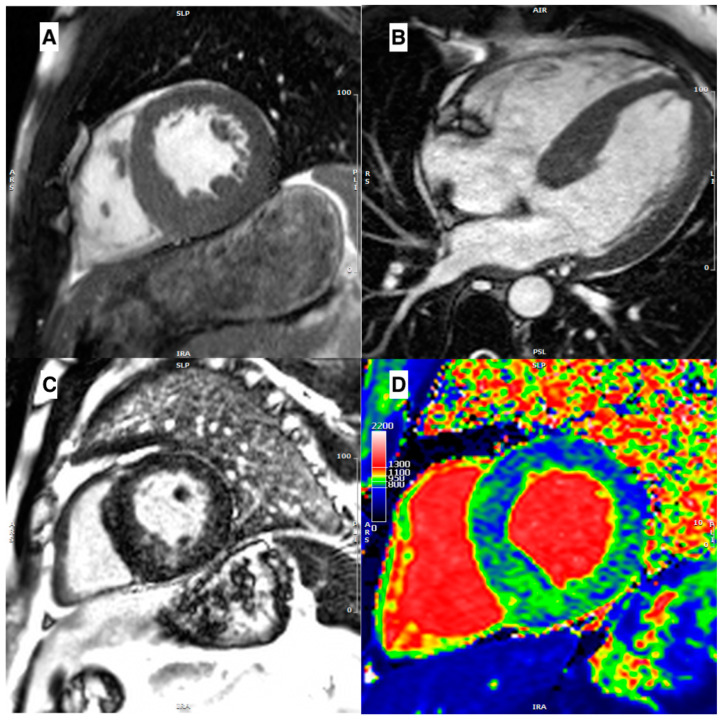
(**A**) Mid-ventricular short axis cine-bSSFP image in telediastole and (**B**) cine-bSSFP 4-chamber image in telediastole, both showing a case of Anderson–Fabry disease with asymmetric septal LV hypertrophy. (**C**) Mid-ventricular short axis LGE image showing mid-wall LGE in inferoseptum and inferior wall. (**D**) Mid-ventricular short axis T1 map showing a diffuse reduction of intra-myocardial T1 values, with T1 pseudo-normalization in segments with LGE.

In ***AFD***, morphological assessment may show generic hypertrophic features, not specific ones for a precise diagnosis. When LVH is present, it is usually concentric, but RV hypertrophy, asymmetric septal hypertrophy (Figure 3A,B), and even LVOT obstruction may be identified, in the same form of a sarcomeric HCM [42]. Therefore, tissue characterization is essential. AFD not only causes remodeling of the LV, but also the LA. Studies suggest that enlargement of the LA and decreased atrial compliance may occur before LVH develops. While patients with HCM tend to have larger LA volumes, a reduction in left atrial function is observed in both conditions [43,44]. Unlike HCM, LVOT obstruction is relatively rare, but it can be present in a subset of patients and unmasked by exercise [45,46].

Although LV hypertrophy is uncommon in all kinds of ***mitochondrial disease***, 17 percent of children with diverse subtypes of mitochondrial myopathies had an associated cardiomyopathy in a large study. Their cardiomyopathy was typically accompanied by concentric hypertrophy and considerable LV volume and function fluctuation [32]. In CPEO/KSS patients, usually, hypertrophy is absent or present as a mild septal hypertrophy. In MELAS/-like LVH is more common as a concentric pattern. In other mitochondrial diseases (such as MEERF), LVH, when present, usually involves interventricular septum [47,48].

### 3.2. Role of Edema

CMR possesses a wide range of tissue characterization techniques that enable differentiation between acute and chronic myocardial damage. Conventionally, the presence of edema is considered an indicator of acute damage. In the clinical setting, myocardial edema is recognized as a distinctive marker of the severity of myocardial injury in cases of acute myocardial infarction [49,50]. The application of edema imaging is not only restricted to acute myocardial infarction, but can also aid in the diagnosis of other cardiac conditions such as sarcoidosis, acute rejection following heart transplantation, acute myocarditis, and stress-induced cardiomyopathy. In clinical practice, the fast spin-echo triple inversion recovery sequence T2-weighted (STIR) imaging technique is utilized to assess myocardial edema. Recently, new parametric mapping techniques have been introduced, which enable measurement of the amount of free water within the myocardium, resulting in a more comprehensive evaluation of myocardial edema. For more information on parametric mapping, please refer to the details provided below.

The application of STIR imaging has revealed a correlation between ***HCM*** and myocardial edema or inflammation, resulting in clinical symptoms such as chest pain, syncope, or elevated troponin T levels [51]. Myocardial hyperintensity is frequently observed on T2-weighted images, often co-localizing with areas of LGE, but may also be present in areas outside of LGE [52].

Melacini et al. have proposed that the observed T2 anomalies in HCM may be associated with ischemia caused by microvascular dysfunction, impaired diastolic relaxation, capillary density mismatch, interstitial fibrosis, and/or myocardial bridging. In this context, ischemia affecting hypertrophic myocardial segments may result in mild intramural damage rather than subendocardial damage. During the acute/subacute phase, T2-weighted hyperintensity may be detectable but could eventually fade, while LGE may persist as a chronic scar [53]. The finding of a close spatial correlation between the area of T2-weighted hyperintensity and the area of hypoperfusion, as detected by the first-pass gadolinium cardiovascular magnetic resonance (CMR) technique, strongly supports the concept that microvascular disease and ischemia are causative factors in the development of edema [54].

Todiere et al. reported T2-weighted hyperintensity in 42% of patients with HCMs, which was the most significant predictor of non-sustained ventricular tachycardia (NSVT) during a 24 h Holter ECG recording. Moreover, patients exhibiting T2-weighted hyperintensity had larger LV mass indices, reduced ejection fractions, and a higher degree of LGE. These findings indicate that, in the advanced stages of HCM, ischemia episodes may be more intense and prolonged than in the early phases, potentially leading to myocardial damage and arrhythmias [55].

### 3.3. Role of Late Gadolinium Enhancement

LGE is the established CMR technique for characterizing cardiac tissue. It differentiates between ischemic and non-ischemic heart diseases by identifying distinct enhancement patterns, and is particularly effective in identifying localized scarring or fibrosis [56]. LGE is more closely associated with pathological hypertrophy and plays a critical role in diagnosing LVH. However, it relies on the suppression of the regular myocardial signal, which can make it challenging to detect extensive myocardial fibrosis.

In ***HCM***, approximately 50% of patients exhibit signal enhancement on LGE images [57]. LGE typically presents mid-wall and patchy enhancement in thickened regions, with a predilection for the anterior and posterior right ventricular (RV) insertion sites (Figure 1D). These sites exhibit plexiform fibrosis of the left ventricle (LV) and RV crossing fibers, although this pattern is not exclusive to HCM (similar findings could be observed in patients with right ventricular hypertrophy). LGE typically affects the interventricular septum, particularly the anteroseptal mid-to-basal regions, and has a noncoronary distribution. In cases of apical aneurysms and end-stage HCM, where the LV wall is thinned, full-thickness (“transmural”) LGE can be present [58].

Numerous studies have shown that LGE is a progressive and rapid phenomenon that is associated with adverse cardiac remodeling. Progression of fibrosis is associated with more severe disease at baseline, characterized by an LGE extent of >8% of LV mass, an indexed LV mass of >100 g/m^2^, maximal wall thickness of ≥20 mm, a left ventricular ejection fraction (LVEF) of ≤60%, and the presence of an apical aneurysm [59].

LGE evaluation is very important in differentially diagnosing HHD; in this latter case, LGE is rarely seen without the above-described characteristics.

Patients with ***CA*** often exhibit a characteristic appearance in LGE images after the administration of gadolinium-based contrast agents (GBCA). This appearance is referred to as “early darkening”, because of a clear darkening of the LV cavity. Some authors attribute this phenomenon to the rapid wash-in/wash-out of GBCA in the blood pool [60]. However, current authors suggest that the early darkening appearance may result from the interaction between a compound with a long T1 (amyloid) and a compound with a short T1 (GBCA) in the blood pool. This results in a faster loss of signal in the LV cavity in LGE sequences, causing the blood and myocardium to null together, leading to very similar signal intensities between the LV cavity and blood pool. Furthermore, a specific global subendocardial RV and LV side “tram-line” LGE is observed, followed by transmural LGE in later stages (Figure 2E,F). CA involves a continuum ranging from minimal LGE to subendocardial to transmural tracking, with increasing amyloid deposition.

In ***AFD***, approximately 50% of patients display the characteristic mid-wall LGE pattern in the basal inferolateral wall. However, other conditions, such as myocarditis and desmosomal diseases, may also present with a similar pattern. Typically, LGE occurs after the development of left ventricular hypertrophy, but it is increasingly being identified in female heterozygotes prior to the onset of hypertrophy. The majority of individuals with LVH will have LGE, while approximately 15% of patients without hypertrophy will also have LGE. As the disease progresses, LGE may become more diffuse (Figure 3C). Furthermore, the presence of increasing fibrosis, as indicated by LGE, is a crucial prognostic factor for malignant ventricular arrhythmias and cardiac prognosis in AFD [61].

In cases of ***mitochondrial disease***, variable LGE expression has been described in the literature. In contrast to MERRF syndrome patients, CPEO/KSS and MELAS patients are more likely to have LGE. Particularly prevalent in CPEO/KSS is a mid-wall LGE in the inferolateral wall, but in MELAS/-like individuals, a mid-wall LGE with extension in the majority of myocardial segments is seen [48].

### 3.4. Role of Parametric Mapping

Newer techniques for parametric mapping, such as T1 and T2 mapping, allow for enhanced tissue characterization using pixel-wise quantitative mappings [62]. The native T1 values, which are obtained prior to administering contrast agents, can be affected by an increase in tissue free water content and can be prolonged by inflammation, edema, or localized and widespread fibrosis [63]. Conversely, tissue iron concentration, lipid deposition (as observed in AFD), and GBCAs can decrease T1 levels [64]. T1 mapping is well-validated for identifying early subtle myocardial alterations in a wide range of cardiac diseases and may aid in distinguishing LVH [65].

Furthermore, quantifying the extracellular volume (ECV) of the myocardium has become an area of increasing interest, as it can serve as a surrogate marker for diffuse interstitial fibrosis. ECV is determined by calculating pre- and post-contrast myocardial and blood T1 measurements, with correction for hematocrit.

T2 mapping, similar to native T1 mapping, represents the overall signal from the intracellular and extracellular myocardial compartments. An elevated T2 value is usually indicative of a higher free water content and is seen in conditions such as ischemia and inflammation. Although T2 mapping is not a primary tool for assessing cardiomyopathy, elevated T2 values have been reported in AFD, indicating an inflammatory component in its pathogenesis [66].

In ***HCM***, using mapping sequences allows clinicians to analyzed further than isolated tissue anomalies. Dass et al. showed longer native T1 values in HCM patients compared to controls, with LGE segments showing the most significant increase [67]. Enhancing this concept, Kato et al. showed that LGE-negative myocardial segments could have longer native T1 values, in particular in hypertrophied segments [68]. Interestingly, Huang et al. reported higher values of both T1 and T2 despite normal wall thickness, indicating that a probable tissue remodeling may precede morphological and functional changes in HCM [69]. In addition, Amano et al. linked elevated T2 values with elevated troponin T and brain natriuretic peptide (BNP) levels, highlighting an acute myocardial injury [70]. Finally, Wang et al., by applying radiomic analysis of native T1 values, differentiated between HCM induced by mutation of MYH7 (β-myosin heavy chain) or MYBPC3 (β-myosin-binding protein C) [71].

While it is not surprising to find higher T1 values in LGE/hypertrophied segments, it must be underlined that native T1 mapping does not always allow for differentiation between HCM and other phenocopies, suggesting that mapping techniques should be merged with clinical, morphological, and conventional sequence information to make a correct diagnosis [72].

Sado et al. demonstrated, as expected, higher ECV values in HCM than in controls [73]. More interesting are the results from Swoboda et al., that found an inverse correlation between ECV values and maximal wall thickness in competitive athletes, and a positive association in HCM patients [74]. It has been proposed that ECV can be used to differentiate between HCM, where hypertrophy is due to extracellular space expansion, and athletic remodeling, where hypertrophy is mediated by a true cellular hypertrophy.

The current authors believe that, although not reported in international recommendations, a modern CMR study should use all weapons for tissue characterization, instead of relying only on LGE analysis. Therefore, use of parametric mapping is recommended when assessing a patient with LVH in addition to, but not as a substitute for, conventional sequences.

***CA*** was one of the first myocardial diseases assessed using mapping sequences. Karamitsos et al. tested the potential role of native T1 mapping in AL CA, showing an area under the curve (AUC) for the detection of definite or possible cardiac involvement of 0.97 [75]. Fontana et al. tested the diagnostic performance of native T1 mapping also in ATTR, showing how T1 was elevated compared with HCM and normal subjects [76]. Baggiano et al. showed that native T1 was associated with high diagnostic accuracy in both AL and ATTR CA (AUC for overall population of 0.93), and suggested cut-off values associated with optimal negative and positive predictive values, in order to exclude or confirm cardiac involvement with non-contrast CMR exams [77]. However, these cut-offs are to be interpreted with caution because it is well known that several factors (patient-related and exam-related) influence T1 values.

The diagnostic performance differences between native T1 and ECV among the various types of CA need additional clarification. Native T1 provides a signal that is a combination of the intracellular and extracellular spaces, while ECV detects the extracellular space directly. In light-chain toxicity in AL CA with acute and fast progression, the native myocardial T1 is greatly influenced by the tissue’s water content. In contrast, ECV levels, which are strongly connected to amyloid accumulation, are often astoundingly high in ATTR, where amyloid deposition is typically more plentiful but more diluted over time than in AL. These differences may explain the significantly greater T1 values in AL over ATTR, as well as the higher ECV values in ATTR versus AL. Additionally, it must be considered that the magnetic properties of amyloid and the altered gadolinium kinetics (see above) may significantly influence the post-contrast T1 values of both the myocardium and the blood pool, resulting in a falsely very high ECV value, especially in ATTR, due to its chronic course. Even if a real significant expansion of the extracellular matrix occurs in CA, before accepting observed ECV values as accurate, it is necessary to take into account the varied behaviors caused by distinct physical features. It is vital to know the causes of very high ECV values in CA, but it is also important to highlight that a new pathognomonic feature of CA in CMR is now available. Native T1 mapping and ECV from a case of AL CA are shown in Figure 2C,D.

Kotecha et al. studied the role of myocardial oedema in CA. They found that T2 was highest in CA, especially in untreated AL patients, next-highest in treated AL patients, and, finally, lowest in ATTR patients [78]. To date, there is a lack of large-scale studies that have evaluated changes in mapping CMR parameters during treatment. However, there is some promising data suggesting that disease-modifying therapies may result in better preservation of functional data, such as global longitudinal strain (GLS), myocardial work index, and myocardial work efficiency, over a 12-month period compared to a cohort not treated with Tafamidis [79]. These data support the use of Tafamidis as a potential treatment for this patient population. A post hoc analysis of 16 patients treated with Patisiran in combination with diflunisal has shown a reduction in CMR-derived ECV, along with a reduction in serum TTR and cardiac biomarkers. However, caution is advised when interpreting these findings [80]. In a prospective cohort study of 33 patients with ATTR CA, Inotersen treatment was found to lead to a reduction in LV mass on CMR after 2 years of treatment [81]. Although these findings are promising, large-scale trials are needed to confirm the ability of these therapies to induce disease regression.

As a result of lipid accumulation, native T1 levels are often lower in patients with ***AFD*** [65]. In the early stages of the disease, there is an accumulation of lipids within the cells, which is typically observed along the inferolateral wall. This accumulation causes a decrease in T1 values in T1 mapping. As the disease progresses, there is a gradual injury to the cells, resulting in extracellular deposition of fibrotic material that can be detected by LGE. At this stage, the native T1 values may appear normal (“pseudo-normalization”) or even elevated [82]. Thompson et al. found that T1 mapping is sensitive and specific regardless of sex, LV shape, and function in individuals with AFD [83]. Moreover, a very intriguing observation is the role of native T1 as an early marker of the disease, Pica et al. showed that the decline of native T1 values precedes LVH in 40% of cases [84]. A diffuse T1 reduction in a case of AFD is shown in Figure 3D. Nordin et al. reported elevated T2 values in inferolateral segments with LGE, and that all patients with LGE had elevated blood troponin levels. Authors have suggested considering AFD not merely as a storage disease, but rather as a chronic inflammatory cardiomyopathy [66]. In addition, T1 and T2 levels may be utilized effectively to monitor response to enzyme replacement treatment (ERT) [85].

In ***mitochondrial disease***, only sporadic and uncommon case reports used mapping methods. The rarity of these diseases explains the dearth of data in this sector; thus, more research is required to assess whether parametric mapping approaches might provide significant data to this subject.

### 3.5. Contractility Assessment

Methods for evaluating myocardial contractility and pathological myofiber disarray are further sophisticated CMR techniques used to evaluate LVH. Myocardial strain imaging has emerged as a sensitive indicator of early subclinical myocardial dysfunction. Feature tracking (CMR-FT), which monitors myocardial boundaries through time on cine images, is thoroughly verified and studied across a broad spectrum of conditions, including the differentiation of LVH.

In ***HCM***, the assessment of myocardial strain provides valuable information about the mechanisms underlying the disease. Studies have shown that functional abnormalities in the myocardium can extend beyond the presence of LGE. Specifically, abnormal intramural systolic strain has been observed in hypertrophied segments of the heart compared to segments without hypertrophy, regardless of whether LGE is present or not [86]. Moreover, a linear correlation between myocardial strain and LGE extent has been shown [87].

Similarly as in echocardiography, patients with *CA* being evaluated with CMR feature tracking typically show a pattern of relative apical sparing.

A study by Williams et al. included 83 patients and compared CMR strain in patients with ***CA*** (45 patients), HCM, and AFD (19 patients each). Their findings showed that patients with CA had significantly lower longitudinal strain compared to both HCM and AFD. The study also assessed the relative regional longitudinal strain ratio (RRSR), which is an alternative parameter used to evaluate the ratio of strain between the basal and apical regions of the heart. The RRSR was found to have significant differences only between CA and AFD [88].

According to a study by Zhao et al., patients with ***AFD*** exhibited impaired LV strain indices at all stages of the disease. The study also found that in the early stages of the disease (prior to the development of LVH), both LV systolic (global peak systolic longitudinal strain) and diastolic (early diastolic longitudinal strain rate) myocardial contractility were impaired compared to normal controls. Furthermore, the study demonstrated that LV longitudinal strain indices were more sensitive than circumferential strain indices in detecting early contractile abnormalities in AFD [89].

## 4. Prognostic Role of CMR in Hypertrophic Phenocopies

The thickness and mass of the left ventricle have prognostic consequences in sarcomeric HCM. Patients with HCM who have massive LVH of 30 mm or more in any thickened LV segment are at the greatest risk for sudden cardiac death (SCD). Consequently, reliable assessment of maximum wall thickness is required for the evaluation of HCM patients. In this sense, echocardiography may underestimate LV wall thickness, with substantial treatment implications [90]. CMR-derived LV mass is the most precise indicator of the whole extent of LVH. However, LV mass may be normal in individuals with HCM, especially when LVH is localized or asymmetric, so it is not an independent predictor of adverse outcomes [91]. An increase in the size of the LA is associated with a higher incidence of morbidity and mortality in patients with cardiovascular disease. It is also regarded as an indicator of an increased risk of SCD and potentially life-threatening arrhythmic events among patients with HCM [92]. LA size could be associated with poor outcome (such as stroke) because it is a predictor of atrial fibrillation (AF) development. In a study involving 653 individuals with low-risk HCM, it was observed that older age, larger LA dimension, and LVOT obstruction were predictive factors for the development of AF [92]. Moreover, Kramer et al. reported that older age, high BMI, moderate or severe MR, history of arrhythmia, increased LA volume, and reduced LA contractile percentage predicted hospitalization for >24 h, electrical cardioversion, ablation, or decision to allow permanent AF [93].

Several prospective outcome studies have shown a correlation between the occurrence of LGE and adverse cardiac events [94,95]. Differentiating LGE presence from its extent has to be given attention. The majority of research has indicated a correlation between LGE and SCD in HCM. However, the reported incidence of LGE is up to 88% in HCM patients; hence, LGE alone would not qualify as a practical risk marker, since an excessive number of patients would be selected for primary preventive ICD. The amount of LGE appeared as a major predictor of SCD in a large multicenter international prospective investigation which included almost 1300 participants [96]. Significant LGE (>15% of LV mass) had a 2-fold increased risk of SCD compared to individuals without LGE, even in the absence of traditional risk factors. Patients lacking LGE, on the other hand, had a benign course. In addition, when LGE extent was combined with the standard SCD risk factors, the amount of LGE reinforced the existing SCD. In addition, Todiere et al. demonstrated that a 10% or higher extent of LGE may identify individuals with a low-to-moderate ESC SCD risk score who are at an elevated risk for heart disease [97].

As a result of these observations, the recent American and European guidelines consider extensive LGE in the evaluation for ICD implantation [12,98]. In particular, according to the latest 2022 ESC guidelines for the prevention of sudden cardiac death, an LGE ≥ 15% in medium- or low-risk patients should be assessed, contributing to strengthening the indication for ICD implantation [98].

Avanesov et al. investigated the prognostic role of ECV in HCM patients, finding that it is a strong predictor (as expected, since LGE extent is a confirmed predictor of prognosis) and performed similarly to the HCM Risk-SCD score in predicting cardiac events [99]. Furthermore, Banypersad et al. demonstrated that both native T1 mapping and ECV are good predictors of mortality in AL CA [100]. Conversely, in ATTR CA, Martinez-Naharro et al. showed that both native T1 and ECV correlate with mortality, but only ECV is an independent predictor of prognosis [101].

CMR has the potential to be a valuable tool for assessing patient characteristics, both before and after septal reduction therapy. According to Spirito et al., CMR can provide high-resolution images and should be routinely used for pre-procedural anatomic assessment of interventricular septum in patients undergoing myectomy [102]. Factors such as the presence of a focal septal bulge, a wide angle of the papillary muscles, and chords to the ventricular septum favor alcohol septal ablation (ASA), whereas midventricular hypertrophy leans toward surgical myectomy. A septal thickness of ≥17 mm is a widely accepted cut-off for safely performing ASA and minimizing the risk of an iatrogenic ventricular septal defect [103]. However, the procedure may not be optimal in cases of severe hypertrophy (>25 mm), possibly due to the need for high-dose alcohol infusion and the subsequent increased risk of complications. Additionally, CMR can be used during follow-up to measure the size and location of the iatrogenic infarct after ASA, and to assess reasons for procedural failure, such as a small or misplaced iatrogenic infarct. Finally, Amano et al. reported a decrease in posterior wall thickness, myocardial mass, and left atrial diameter, suggesting a possible ASA effect in remote and global myocardium during follow-up [104].

## 5. Conclusions

CMR is an available diagnostic tool permitting to identify hypertrophic phenotypes. The recent clinical application of T1 and T2 mapping has increased diagnostic accuracy. The quantitative analysis of mapping could be used as a surrogate marker of therapy efficacy in CA and AF diseases. However, large trials are needed to use mapping as a follow-up tool to guide therapy. Today, the presence and extent of LGE is a further independent prognostic factor in the stratification of sarcomeric HCM.

## Figures and Tables

**Table 1:** CMR findings in HCM phenocopies.

Disease	Morphological Features	Late Gadolinium Enhancement	Myocardial T1	ECV
Sarcomeric HCM, Pediatric HCM	Several different patterns of hypertrophy Apical aneurysm Crypts Papillary\mitral abnormalities SAM\LVOT obstruction	Mid-wall in hypertrophied segments	Increased only in fibrotic areas	Increased only in fibrotic areas
Amyloidosis	Concentrical pseudo-hypertrophy Thickened LA wall Pericardial effusion	Low-difference signal intensity blood-cavity Diffuse subendocardial	Diffusely increased	Diffusely increased
Fabry disease	Diffuse hypertrophy (80%), asymmetrical	Inferolateral mid-wall (only in late stage)	Diffusely decreased (increased in LGE areas)	Diffusely decreased (increased in LGE areas)
Hypertensive heart disease	Usually, concentric hypertrophy with wall thickness not exceeding 16 mm Rarely, SAM/LVOT obstruction	No LGE	Normal or slightly increased	Normal or slightly increased
Mitochondrial cytopathies	Asymmetric (septal) or concentric hypertrophy	Mid-wall LGE in the inferolateral wall or extending in the majority of myocardial segments	Increased, particularly in segments with LGE	Increased, particularly in segments with LGE
Glycogen storage disorders	Diffuse or focal myocardial hypertrophy	Patchy mid-wall LGE in septum and insertion point or extending diffusely	Increased	Increased

## Data Availability

Data sharing not applicable.

## Supplementary Materials

The following supporting information can be downloaded at: https://www.mdpi.com/article/10.3390/jcm12103481/s1. Supplemental Video: cine-bSSFP 3-chamber video showing a case of HCM with asymmetrical septal hypertrophy. Signal void in LV outflow-tract during systole is noted as a sign of obstruction. Moreover, a SAM is evident with characteristic inferolaterally-directed mitral regurgitation.
